# Integrin CD11b mediates locus coeruleus noradrenergic neurodegeneration in a mouse Parkinson’s disease model

**DOI:** 10.1186/s12974-020-01823-3

**Published:** 2020-05-06

**Authors:** Liyan Hou, Xingyue Qu, Xiaofei Qiu, Ruixue Huang, Xiulan Zhao, Qingshan Wang

**Affiliations:** 1 School of Public Health, Dalian Medical University, Dalian, 116044 China; 2 Department of Clinical Nutrition, Second Affiliated Hospital of Dalian Medical University, Dalian, 116023 China; 3 Qingdao Municipal Center for Disease Control & Prevention/Qingdao Institute of Preventive Medicine, Qingdao, 266033 China; 4 School of Public Health, Cheeloo College of Medicine, Shandong University, Jinan, 250012 China; 5 National-Local Joint Engineering Research Center for Drug-Research and Development (R & D) of Neurodegenerative Diseases, Dalian Medical University, Dalian, 116044 China

**Keywords:** Integrin, Noradrenergic neurodegeneration, NLRP3 inflammasome, Microglial activation, Oxidative stress

## Abstract

**Background:**

The loss of locus coeruleus noradrenergic (LC/NE) neurons in the brainstem is reported in multiple neurodegenerative disorders, including Parkinson’s disease (PD). However, the mechanisms remain unclear. Strong evidence suggested that microglia-mediated neuroinflammation contributes to neurodegeneration in PD. We recently recognized integrin CD11b, the α-chain of macrophage antigen complex-1 (Mac-1, also called CR3), as a key regulator for microglial activation. However, whether CD11b is involved in LC/NE neurodegeneration in PD remains to be investigated.

**Methods:**

LC/NE neurodegeneration and microglial activation were compared between wild type (WT) and CD11b KO mice after treated with paraquat and maneb, two pesticides that widely used to create PD model. The role of NLRP3 inflammasome in CD11b-mediated microglial dysfunction and LC/NE neurodegeneration was further explored. LC/NE neurodegeneration, microglial phenotype, and NLRP3 inflammasome activation were determined by using Western blot, immunohistochemistry, and RT-PCR technologies.

**Results:**

Paraquat and maneb co-exposure elevated the expressions of CD11b in the brainstem of mice, and CD11b knockout significantly reduced LC/NE neurodegeneration induced by paraquat and maneb. Mitigated microglial activation and gene expressions of proinflammatory cytokines were also observed in paraquat and maneb-treated CD11b^−/−^ mice. Mechanistically, CD11b-mediated NLRP3 inflammasome activation contributes to paraquat and maneb-induced LC/NE neurodegeneration. Compared with WT controls, CD11b deficiency reduced paraquat and maneb-induced NLRP3 expression, caspase-1 activation, and interleukin-1β production in mice. Furthermore, inhibition of NLRP3 inflammasome by glybenclamide, a sulfonylurea inhibitor of NLRP3 inflammasome, was found to be able to suppress microglial proinflammatory activation and nuclear factor-κB activation induced by paraquat and maneb. Moreover, reduced reactive oxygen species production, NADPH oxidase, and inducible nitric oxide synthase expressions as well as 4-hydroxynonenal and malondialdehyde levels were detected in combined glybenclamide and paraquat and maneb-treated mice compared with paraquat and maneb alone group. Finally, we found that glybenclamide treatment ameliorated LC/NE neurodegeneration and α-synuclein aggregation in paraquat and maneb-treated mice.

**Conclusion:**

Our findings suggested that CD11b mediates LC/NE neurodegeneration through NLRP3 inflammation-dependent microglial proinflammatory activation in a two pesticide-induced mouse PD model, providing a novel insight into the immune pathogenesis of LC/NE neuronal damage in related disorders.

## Background

The locus coeruleus noradrenergic (LC/NE) neurons in the upper dorsolateral pontine tegmentum contain more than half of the brain’s cellular capacity for synthesizing norepinephrine [1]. Axons of LC/NE neurons are distributed widely throughout the brain, suggesting a prominent role of LC/NE neurons in the normal function of central nervous system (CNS) [1, 2]. The degeneration of LC/NE neurons is detected in multiple neurodegenerative diseases, such as Alzheimer’s disease (AD) and Parkinson’s disease (PD) [3, 4]. Postmortem studies demonstrated that the loss of LC/NE neurons is greater and may be earlier than that of nigral dopaminergic neurons in patients with PD [5, 6]. Furthermore, lesion of LC/NE neurons by *N*-(2-chloroethyl)-*N*-ethyl-2-bromobenzylamine (DSP-4), a selective LC/NE neurotoxicant, exacerbates dopaminergic neurodegeneration and motor deficits in mouse PD models generated by lipopolysaccharide (LPS) or 1-methyl-4-phenyl-1,2,3,6-tetrahydropyridine (MPTP) [7–9], highlighting its importance in the pathogenesis of PD. However, the mechanisms of LC/NE neurodegeneration remain unclear.

Integrin CD11b is the α-chain of macrophage antigen complex-1 (Mac1, also called CD11b/CD18 or CR3) and presents with high levels in innate immune cells, including microglia [10–12]. Although CD11b is well-documented to regulate phagocytosis and migration of microglial cells [11, 13], studies revealed that CD11b can also recognize a variety of stimuli to mediate neuroinflammation and neurodegeneration [14–16]. Zhang et al. reported that microglial CD11b is critical for the neurotoxicity induced by β-amyloid (Aβ), the main component of senile plaques in AD, since CD11b knockout significantly attenuates Aβ-induced microglial activation, superoxide production, and neurodegeneration in both in vitro and in vivo conditions [17]. Furthermore, Czirr et al. reported that ablation of CD11b in human amyloid precursor protein–transgenic mice decreased accumulation of Aβ in the brain [18]. In a mouse stroke model induced by middle cerebral artery occlusion (MCAO), genetic inactivation of CD11b also reduced blood brain barrier permeability and infarct volume [19]. We recently found that α-synuclein, a protein that accumulates in Lewy bodies and Lewy neurites in PD, interacts with CD11b to stimulate microglial activation and superoxide production in microglial cells [20]. More importantly, CD11b deficiency markedly suppresses microglial activation and dopaminergic neurodegeneration in paraquat and maneb-induced mouse PD model [21], suggesting that CD11b activation is involved in the pathogenesis of PD. However, whether CD11b contributes to LC/NE neurodegeneration remains to be investigated.

In the present study, we aimed to investigate the role of CD11b in LC/NE neurodegeneration by using a mouse PD model induced by paraquat and maneb co-exposure (referred to subsequently as P + M). We found that P + M elevated CD11b expression and genetic deletion of CD11b in mice significantly ameliorated P + M-induced LC/NE neurodegeneration. Mechanistically, CD11b-mediated NLRP3 inflammasome activation and subsequent microglial proinflammatory activation as well as oxidative damage contributed to LC/NE neurodegeneration in paraquat and maneb-treated mice. Our findings provide a novel insight for the pathogenesis of LC/NE neurodegeneration in PD.

## Materials and methods

### Animal dosing

Male wild type (WT, C57BL/6) and CD11b^−/−^ mice were randomly divided into 2 groups, i.e., control, P + M. Mice in P + M group were administrated (i.p) with combined paraquat (10 mg/kg) and maneb (30 mg/kg) for consecutive 6 weeks (twice per week) according to our previous report [22]. Mice in control group received an equivalent volume of 0.9% saline. Housing and breeding of animals were performed strictly with Dalian Medical University’s Guide for the Care and Use of Laboratory Animals. All animal procedures and their care were carried out in accordance the National Institute of Health Guide for the Care and Use of Laboratory Animals and were approved by the Institutional Animal Care and Use Committee of Dalian Medical University.

### Glybenclamide treatment

Glybenclamide (1 mg/kg) was administrated to mice 30 min before P + M co-exposure for consecutive 6 weeks (twice per week). The chosen of dose of glybenclamide was based on previous report [23].

### Immunohistochemistry

Immunohistochemistry was performed as described previously [24, 25]. Briefly, mice (*n* = 6–8 in each group) were perfusion using 4% paraformaldehyde, and the brains were sectioned into coronal slices (30 μm). The sections were stained with primary antibodies targeting tyrosine hydroxylase (TH, 1:1000; EMD Millipore Corporation, Billerica, MA, USA) or ionized calcium binding adaptor molecule-1 (Iba1, 1:2000; Wako Chemicals, Richmond, VA, USA), followed by treatment with biotin-labeled secondary antibodies and Vectastain ABC reagents. The bound complex was visualized using 3,3′-diaminobenzidine. The densities of the Iba-1 in the LC were measured using the ImageJ software based on our previous method [26].

The number of TH-immunoreactive (THir) neurons in the LC region was visually counted under a microscope (× 200), as described previously [27]. The boundary of LC was outlined under magnification of the × 4 objective as per the atlas [28]. Every three sections from the rostral of a series of 36 sections that cover the entire extent of LC were selected for counting [22].

### Real-time PCR analysis

Mice (*n* = 5–6 in each group) were perfused with PBS only and then scarified. The brainstems of mice were quickly dissected based on the atlas [28] and then were equally divided into two parts for real-time PCR and biochemical analysis, respectively, as described previously [29, 30]. Total RNA was extracted with RNeasy Mini kit (Qiagen, Germantown, MD, USA) and reverse transcribed with an oligodT primer. Real-time PCR amplification was performed using SYBR Premix Ex Taq^TM^ II (Takara Bio Inc. Kusatsu, Shiga, Japan) and Takara Thermal Cycler Dice™ Real Time System according to manufacturer’s protocols. The PCR conditions were 95 °C for 10 s, 55 °C for 30 s, and 72 °C for 30 s for 40 cycles. Relative mRNA gene levels were normalized to the GAPDH mRNA level, and relative expressions were determined by the comparative Ct method [22].

### Western blot analysis

The brainstem samples (*n* = 6 in each group) were homogenized with a homogenizer (10,000–15,000 rpm, 10 s) at 4 °C in cold RIPA lysis buffer containing inhibitors of proteinase and phosphatase as described previously [21, 31]. After centrifugation at 4 °C (3000 rpm, 10 min), the supernatant was separated, and protein content was estimated by using commercial BCA^TM^ Protein assay Kits (Pierce Biotechnology, Inc.). Equal amounts of protein were separated by 4–12% Bis-Tris-polyacrylamide electrophoresis gel and transferred to polyvinylidenedifluoride membranes. The membranes were incubated with primary antibodies against NLRP3 (1:1,000; Abcam, Cambridge, MA, USA), caspase-1 (1:1,000; Abcam, Cambridge, MA, USA and 1:500, Santa Cruz Biotechnology, Dallas, TX, USA), interleukin-1 β (Il-1β; Santa Cruz Biotechnology, Dallas, TX, USA), inducible nitric oxide synthase (iNOS, BD Transduction Laboratories, San Jose, CA, USA), phosphorylated NF-κB, NF-κB (1:1000; Cell Signaling Technology, Danvers, MA, USA), 4-hydroxynonenal (4-HNE, 1:1000; Abcam, Cambridge, MA, USA), p47^phox^ (1:1000; EMD Millipore, Temecula, CA, USA), gp91^phox^ (1:1000; BD Transduction Laboratories, San Jose, CA, USA), and GAPDH (Abcam, Cambridge, MA, USA) overnight at 4 °C and followed by horseradish peroxidase-linked anti-rabbit IgG for 2 h at 25 °C. ECL reagents (Biological Industries, Cromwell, CT, USA) were used as a detection system.

### IL-1β assay

The brainstem tissues were homogenized and centrifuged at 10,000×*g* for 10 min at 4 °C. The levels of IL-1β (lengton Inc, Shanghai, China) in the collected supernatant were determined spectrophotometrically respectively with commercial kits according to the manufacturer’s instructions.

### Statistical analysis

All values were expressed as mean ± SEM. Shapiro–Wilk and Bartlett’s tests were used to analyze the normal distributions and equal variances, respectively. Statistical analysis between two groups was performed using Student’s *t* test when the distribution was normal; otherwise, the non-parametric test was used. The statistical comparisons for multiple groups were analyzed by one-way or two-way ANOVA (treatment/genotype as the independent factors) when the distribution was normal; otherwise, the non-parametric test was used. When ANOVA showed significant differences, pair-wise comparisons between means were tested by Tukey’s post hoc testing for variance homogeneity data or Tamhane’s T2 testing for variance data. *p* < 0.05 was considered to be statistical significant.

## Results

### CD11b mediates P + M-induced LC/NE neurodegeneration in mice

**Fig. 1:**
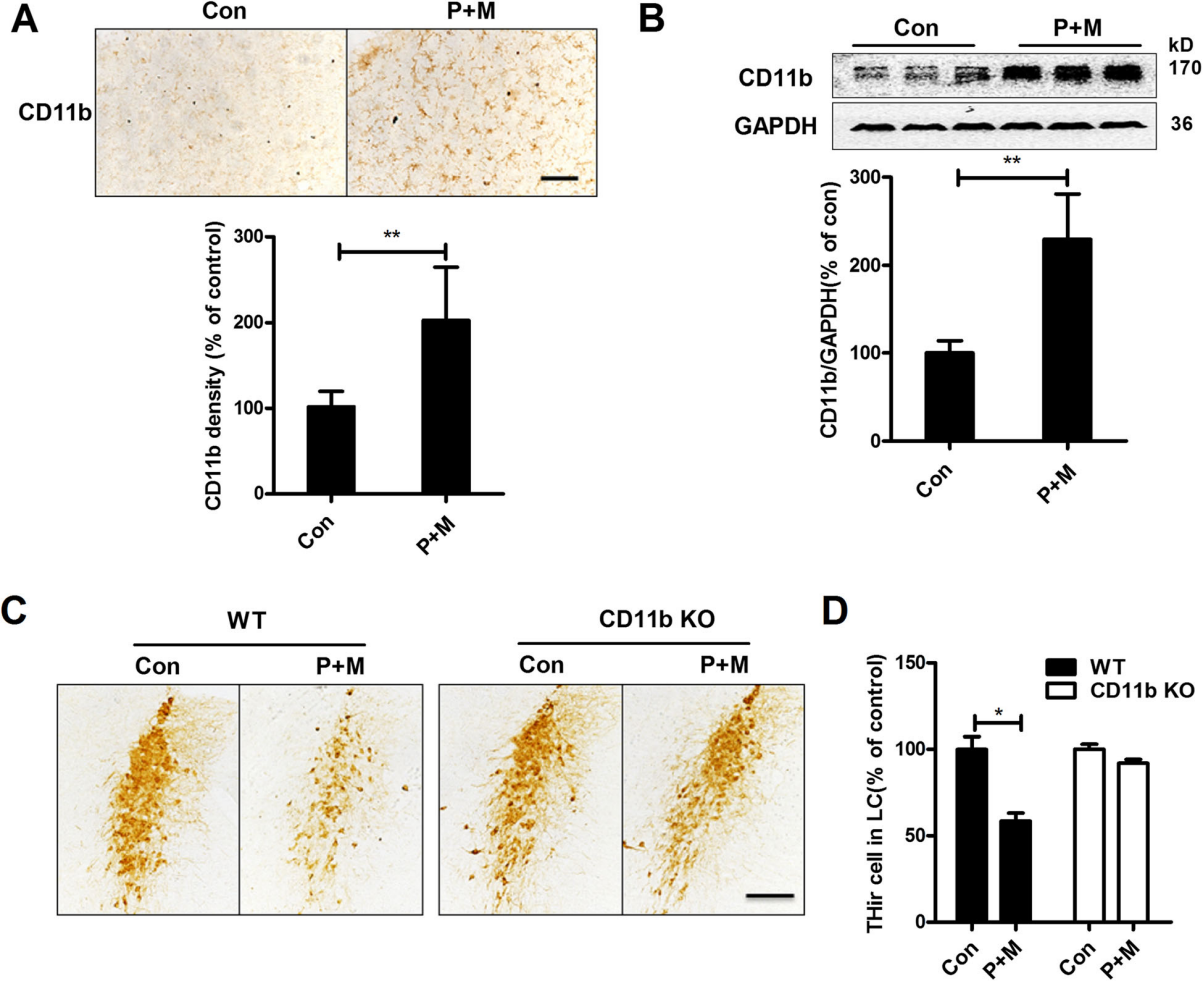
CD11b mediates P + M-induced LC/NE neurodegeneration in mice. **a** CD11b was immunostained with anti-CD11b antibody in the brainstem of P + M-treated mice, and the density of CD11b immunoreactivity was quantified. **b** The expressions of CD11b in the brainstem were determined by Western blot, and the band density of blots was quantified. **c** LC/ NE neurons were immunostained with anti-TH antibody in P + M-treated WT and CD11b KO mice, and the representative images were shown. **d** The number of THir neurons in the LC was counted. *n* = 6 and 8 for WT and CD11b KO group, respectively. **p < 0.01. Scale bar = 100 μm

To determine whether CD11b contributes to LC/NE neurodegeneration in PD, we initially determined the expressions of CD11b in P + M-induced mouse PD model. Immunohistochemistry was performed by using anti-CD11b antibody in saline and P + M-treated mice. Results showed that compared with vehicle controls, P + M exposure significantly increased the expressions of CD11b in the brainstem of mice (Fig. 1**a**). Quantitative analysis of CD11b density supported these observations (Wilcoxon *W* = 45.000, *Z* = − 3.576, *p* < 0.01; Fig. 1a). The elevated expressions of CD11b by P + M treatment were further confirmed by Western blot (*t* = − 7.220, *p* < 0.01; Fig. 1b).

Next, CD11b-deficient mice were used to determine whether CD11b plays a role in P + M-induced LC/NE neurodegeneration. Immunohistochemical staining using anti-TH antibody was performed in the LC, and the number of THir neurons was counted. Consistent with our previous reports [22, 30], a significant decrease of THir neurons in the LC in WT mice after 6 weeks of P + M exposure was observed, indicating LC/NE neurodegeneration (*F*_(3,24)_ = 16.532, *p* < 0.01; Fig. 1 c and d). Interestingly, P + M-induced loss of LC/NE neurons was significantly attenuated in CD11b^−/−^ mice (Fig. 1 c and d).

### CD11b deficiency suppresses P + M-induced microglial activation and gene expression of proinflammatory factors in mice

**Fig. 2:**
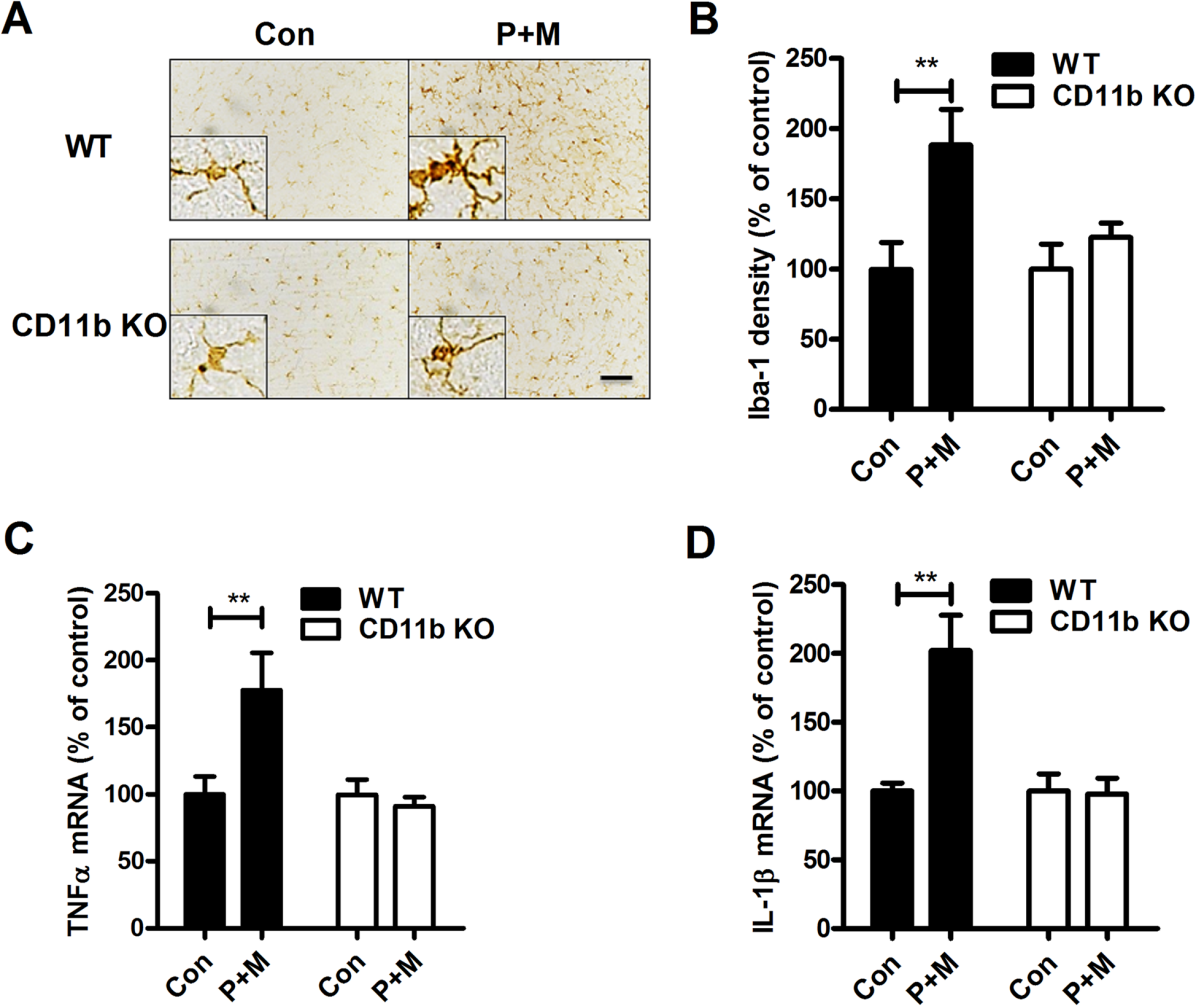
Genetic deletion of CD11b suppresses P + M-induced microglial activation in mice. **a** Microglial cells in the brainstem of P + M-treated WT and CD11b KO mice were immunostained with antibody against Iba-1, and the representative images were shown. Activated microglia are characterized by enlarged cell bodies and high staining density. **b** The density of Iba-1 immunostaining in hippocampus was quantified. **c**, **d** The gene expressions of TNFα (**c**) and IL-1β (**d**) were determined in the brainstem of mice by using RT-PCR. *n* = 6 for each group. *p < 0.05. **p < 0.01. Scale bar = 50 μm

To verify whether the attenuated LC/NE neurotoxicity in P + M-treated CD11b^−/−^ mice was associated with reduced microglial activation, microglia were stained with antibody against Iba-1, a marker of known to upregulate during microglial activation. Activated microglia exhibiting hypertrophied morphology, elevated Iba-1 expression, and increased density of Iba-1 immunostaining in the LC were observed in P + M-treated WT mice (Fig. 2 a and b). In contrast, microglia in P + M-injected CD11b^−/−^ mice revealed ramified resting morphology and similar levels of Iba-1 expression as observed in saline-injected CD11b^−/−^ mice (Fig. 2 a and b).

Activated microglia will release proinflammatory factors, resulting in neuronal damage. The gene expressions of TNFα and IL-1β in the brainstem of both CD11b^−/−^ and WT mice after P+M treatment were determined. Figure 2 c and d showed that P + M exposure greatly increased gene expressions of TNFα (Kruskal Wallis *H* test: *H*(3) = 9.887, *p* = 0.02) and IL-1β (Kruskal Wallis *H* test: H(3) = 12.492, *p* = 0.006) in WT mice, but their expressions were significantly attenuated in CD11b^−/−^ mice. Together, these data demonstrate that CD11b contributes to P + M-induced LC/NE neurodegeneration by augmenting microglia-mediated neuroinflammation.

### CD11b deficiency diminishes P + M-induced NLRP3 inflammasome activation in mice

**Fig. 3:**
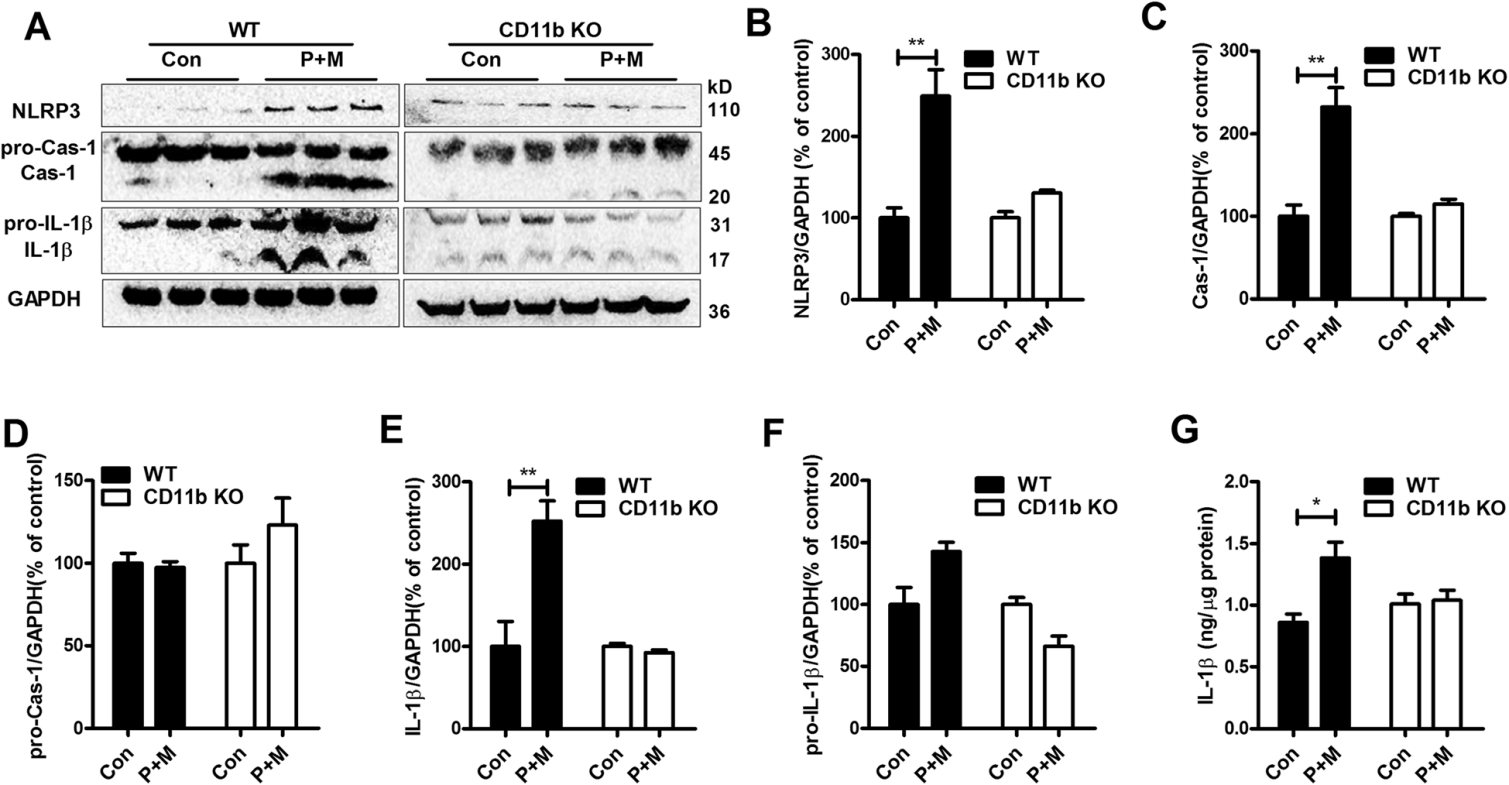
Genetic deletion of CD11b attenuates P + M-induced activation of NLRP3 inflammasome in mice. **a** The expressions of NLRP3, pro-caspase-1, caspase-1 p10, pro-IL-1β, and IL-1β in the brainstem of P + M-treated WT and CD11b KO mice were detected by using Western blot, and the representative blots were shown. GAPDH was used as an internal control. **b**–**f** The band density of NLRP3 (**b**), pro-caspase-1 (**c**), caspase-1 (**d**), pro-IL-1β (**e**), and mature IL-1β (**f**) was quantified. *n* = 6 for each group. **g** The levels of IL-1β in the brainstem of mice were measured by using a commercial ELISA kit. *p < 0.05. **p < 0.01

Previous studies indicated that the activation of nod-like receptor family pyrin domain-containing 3 (NLRP3) inflammasome is a key factor to regulate microglial activation in neurodegenerative diseases [32, 33]. To explore whether NLRP3 inflammasome is involved in CD11b-mediated microglial activation, the effects of CD11b on activation of NLRP3 inflammasome were determined in mice treated with P + M. As illustrated in Fig. 3a–f, P + M exposure resulted in elevation of NLRP3 expression (*F*_(3,20)_ = 15.897, *p* < 0.01), caspase-1 activation (*F*_(3,20)_ = 21.449, *p* < 0.01) and IL-1β maturation (*F*_(3,20)_ = 15.465, *p* < 0.01) in the brainstem of WT mice, although the expressions of pro-capase-1 and pro-IL-1β remained unchanged. ELISA assay also showed an increased content of IL-1β in P + M-treated WT mice compared with vehicle controls (Kruskal Wallis *H* test: *H*(3) = 8.002, *p* = 0.046; Fig. 3g), indicating NLRP3 inflammasome activation. In contrast, in CD11b^−/−^ mice, P + M-induced activation of NLRP3 inflammasome was markedly reduced by showing similar levels of NLRP3 expression, caspase-1 activation, and IL-1β production between P + M-treated mice and vehicle controls (Fig. 3a–g).

### Inhibition of NLRP3 inflammasome by glybenclamide blocks P + M-induced microglial proinflammatory activation

**Fig. 4:**
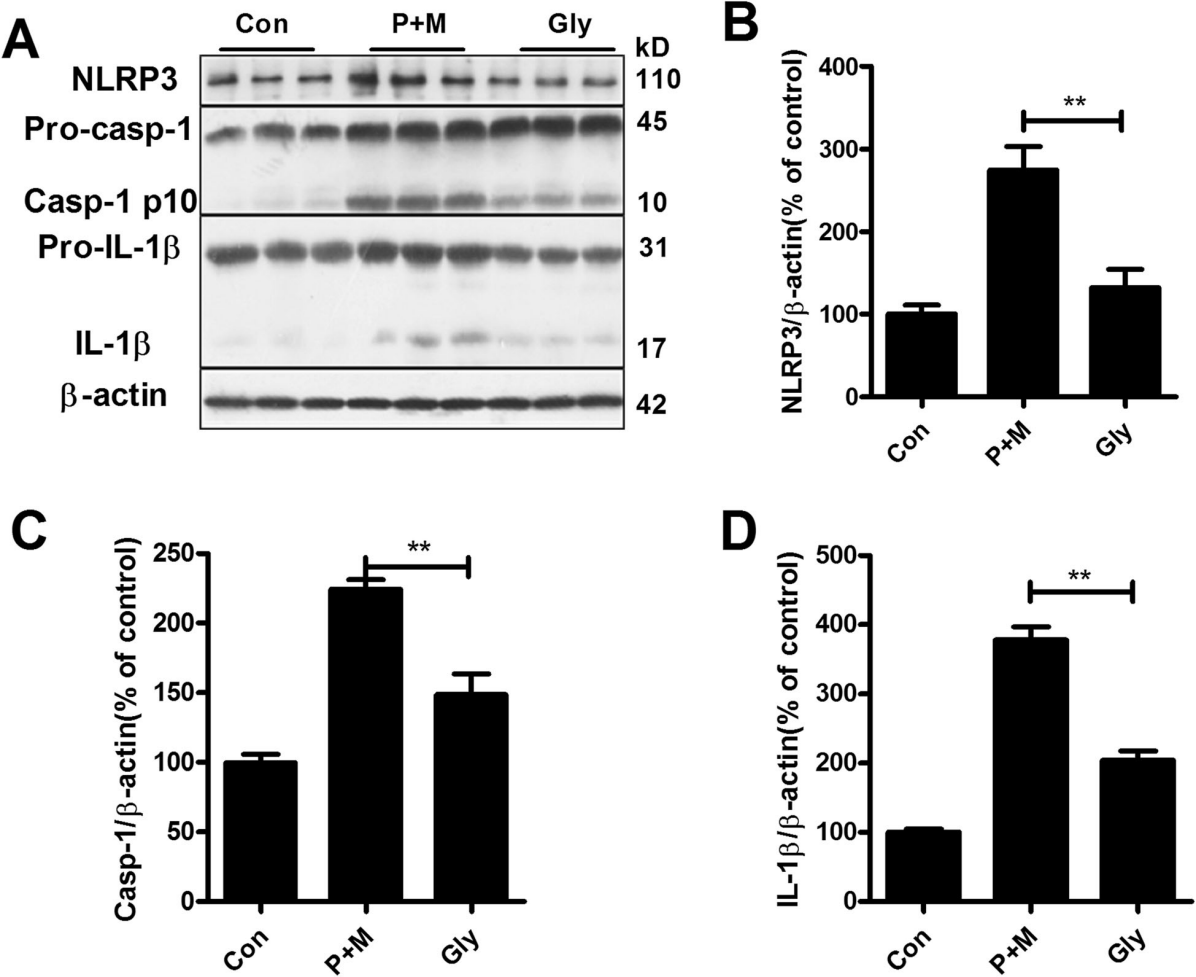
Glybenclamide suppresses NLRP3 inflammasome activation in P + M-treated mice. **a** The expressions of NLRP3, caspase-1, and IL-1β in the brainstem of P + M-treated mice with or without glybenclamide (Gly) were detected by using Western blot, and the representative blots were shown. GAPDH was used as an internal control. **b**–**d** The band density of NLRP3 (**b**), caspase-1 p10 (**c**), and IL-1β (**d**) was quantified. *n* = 6 for each group. *p < 0.05. **p < 0.01

**Fig. 5:**
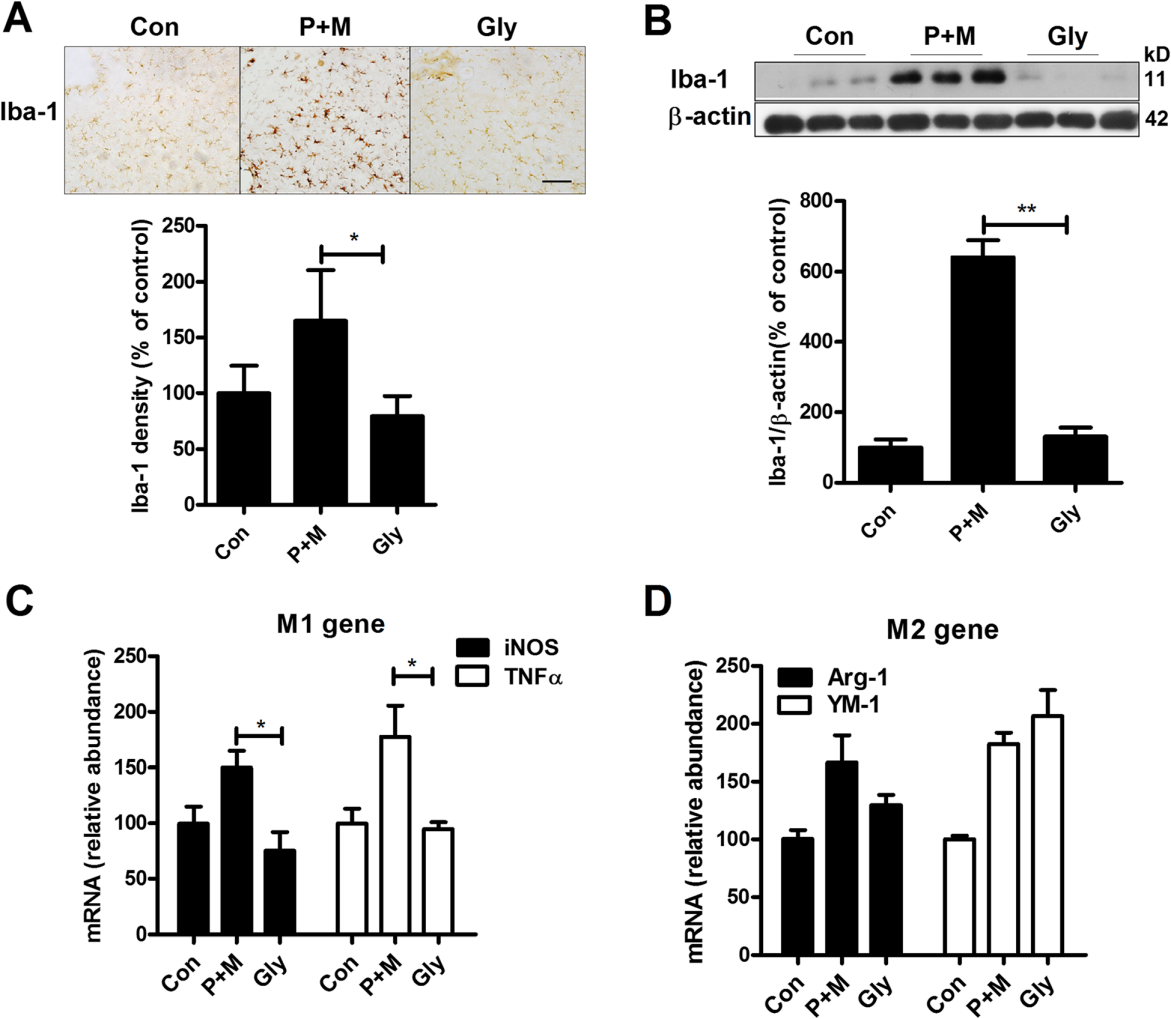
Glybenclamide abrogates microglial proinflammatory activation in the brainstem of P + M-treated mice. **a** Microglial cells in the brainstem of P + M-treated mice with or without glybenclamide pre-treatment were immunostained with anti-Iba-1 antibody, and the density of Iba-1 immunostaining was quantified. **b** The expressions of Iba-1 in the brainstem were determined by Western blot, and the band density of blots was quantified. **c**, **d** The gene expressions of M1 (iNOS and TNFα) and M2 (Arg-1 and YM-1) markers were determined in hippocampus by using RT-PCR. *n* = 5–6 for each group. *p < 0.05. **p < 0.01. Scale bar = 50 μm

Subsequently, we determined the effects of inhibition of NLRP3 inflammasome on P + M-induced microglial activation. Glybenclamide, a widely used sulfonylurea inhibitor of NLRP3 inflammasome, was administrated to mice prior to P + M treatment. Figure 4 revealed that glybenclamide treatment significantly decreased NLRP3 expression (*F*_(2,15)_ = 18.041, *p* < 0.01), caspase-1 activation (*F*_(2,15)_ = 39.052, *p* < 0.01), and IL-1β production (Kruskal Wallis *H* test: *H*(2) = 15.158, *p* = 0.001) in the brainstem in P + M-treated mice, indicating inhibition of NLRP3 inflammasome by glybenclamide. Then, microglial activation was examined in P + M-treated mice with or without glybenclamide pre-treatment. As seen in Fig. 5a, microglia in the LC in P + M-treated mice displayed hypertrophic morphology and increased Iba-1 immunostaining density, which was significantly recovered by glybenclamide. Analysis of Iba-1 immunostaining density and protein expression supported the morphological observation (Fig. 5 a and b).

Microglial activation can be polarized into “classical” (M1) and “alternative” (M2) phenotypes that produce detrimental and beneficial effects, respectively [34]. qPCR analyses revealed that glybenclamide suppressed transcription levels of M1 genes iNOS (Kruskal Wallis *H* test: *H*(2) = 7.833, *p* = 0.02) and TNFα (*F*_(2,14)_ = 5.954, *p* = 0.013) but failed to interfere with M2 genes (Arg-1 and YM-1) in the brainstem in P + M-treated mice, indicating that glybenclamide blocks microglial proinflammatory activation (Fig. 5 c and d).

### Glybenclamide mitigates P + M-induced activation of NF-κB pathway

**Fig. 6:**
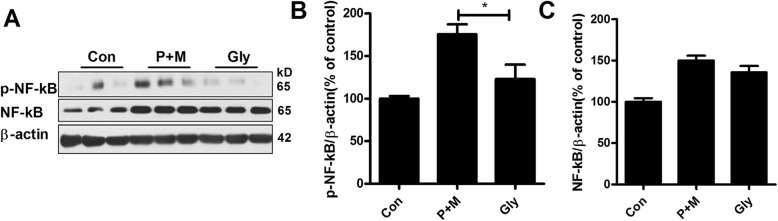
Glybenclamide inhibits P + M-induced NF-κB activation in mice. The expressions of phosphorylated and total NF-κB were determined in the brainstem of P + M-treated mice with or without glybenclamide by Western blot using specific antibodies, and the density of blots was quantified. *n* = 6 for each group. *p < 0.05. **p < 0.01

Strong evidence suggested that the activation of NF-κB signaling is essential for proinflammatory factors production and microglial proinflammatory activation [35, 36]. Consistent with microglial activation, P + M exposure elevated the levels of phosphorylated NF-κB in the brainstem of mice compared with vehicle controls. Interestingly, glybenclamide treatment significantly reduced P + M-induced NF-κB phosphorylation in mice (*F*_(2,15)_ = 10.544, *p* = 0.001; Fig. 6 a and b). Glybenclamide failed to interfere with the expressions of total NF-κB, although P + M also elevated the total levels of NF-κB in mice (Fig. 6 a and c). These results suggested that inhibition of NLRP3 inflammasome by glybenclamide decreased activation of NF-κB signaling in P + M-related mice.

### Glybenclamide mitigates P + M-induced oxidative stress in mice

**Fig. 7:**
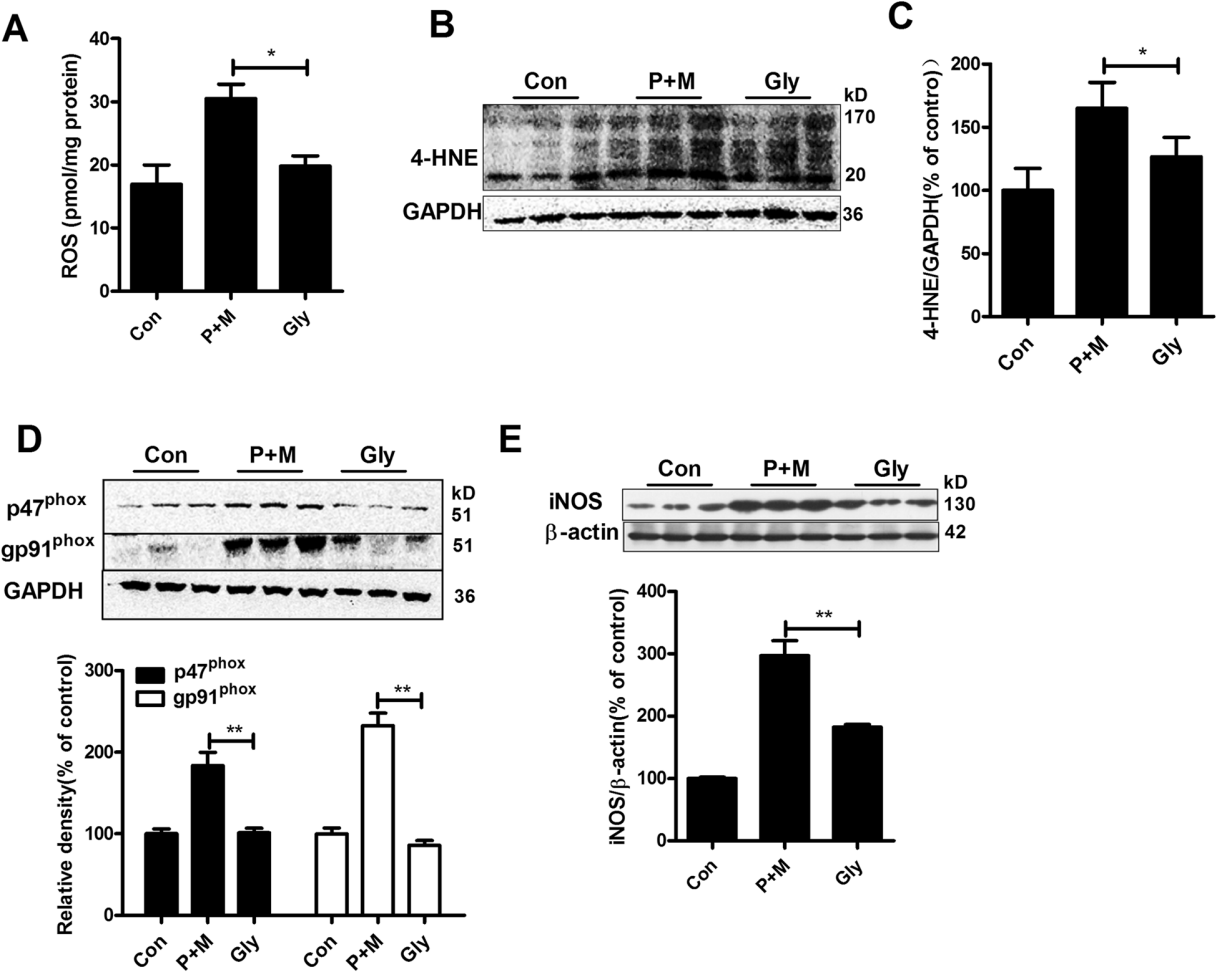
Glybenclamide mitigates P + M-induced oxidative stress in mice. **a** The production of ROS was determined by using DC-FDA probe. *n* = 4. **b**, **c** The expressions of 4-HNE were determined in the brainstem of P + M-treated mice with or without glybenclamide by Western blot, and the density of blots was quantified. **d**, **e** The expressions of NOX2 subunits (gp91^phox^ and p47^phox^) and iNOS were determined in the brainstem of P + M-treated mice with or without glybenclamide by Western blot using specific antibodies, and the density of blots was quantified. *n* = 6 for each group. *p < 0.05. **p < 0.01

Microglial activation, especially proinflammatory activation, produces not only proinflammatory cytokines but also ROS, resulting in oxidative stress in microenvironment [25, 37]. In agreement with inhibition of microglial proinflammatory activation, glybenclamide treatment decreased the production of ROS in the brainstem of P + M-injected mice (Kruskal Wallis *H* test: *H*(2) = 7.538, *p* = 0.023; Fig. 7a). P + M-induced increase of contents of 4-hydroxynonenal (4-HNE), one marker for oxidative stress, was also mitigated by glybenclamide in mice (Fig. 7 b and c).

NADPH oxidase (NOX2) and iNOS are two main enzymes responsible for oxidative stress in microglia-mediated neuroinflammation [38, 39]. To determine whether NOX2 and iNOS are involved in the inhibitory effects of glybenclamide on oxidative stress, the expressions of NOX2 and iNOS were determined. As shown in Fig. 7 d and e, P + M exposure elevated the expressions of p47^phox^ (*F*_(2,15)_ = 20.834, *p* < 0.01) and gp91^phox^ (*F*_(2,15)_ = 61.589, *p* < 0.01), two subunits of NOX2, as well as iNOS (*F*_(2,15)_ = 46.929, *p* < 0.01) in the brainstem of mice, which were significantly reduced by glybenclamide.

### Glybenclamide ameliorates P + M-induced LC/NE neurodegeneration and α-synuclein aggregation in mice

**Fig. 8:**
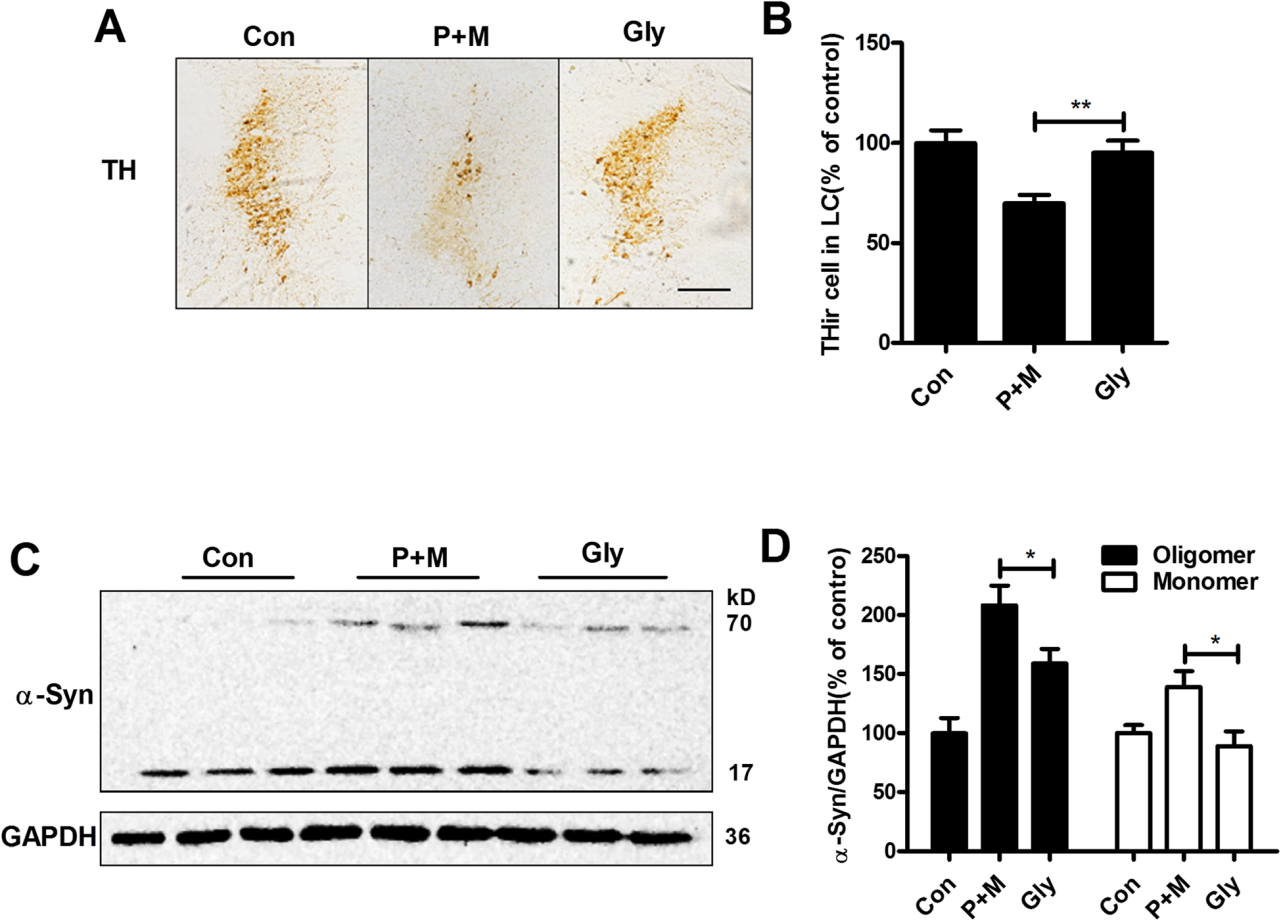
Glybenclamide ameliorates P + M-induced LC/NE neurodegeneration and α-synuclein aggregation in mice. **a** LC/NE neurons were immunostained with anti-TH antibody in P + M-treated mice with or without glybenclamide, and the representative images were shown. **b** The number of THir neurons in the LC was counted. **c** The expression of α-synuclein in the brainstem of P + M-treated mice with or without glybenclamide was detected by using Western blot, and the representative blots were shown. GAPDH was used as an internal control. **d** The band density of blots was quantified. *n* = 6 for each group. *p < 0.05, **p < 0.01

To determine whether inhibition of NLRP3 inflammasome by glybenclamide was able to protect LC/NE neurons, immunohistochemistry using anti-TH antibody was performed in LC, and the number of THir neurons was recorded in P + M-intoxicated mice with or without glybenclamide pre-treatment. As shown in Fig. 8a, compared with P + M alone group, a high number of THir neurons were observed in combined glybenclamide and P + M-treated mice. Quantitative analysis revealed a 25.4% protection afforded by glybenclamide compared with P + M alone group (*F*_(2,15)_ = 8.331, *p* = 0.004; Fig. 8b). In agreement with LC/NE neuroprotection, glybenclamide also reduced α-synuclein aggregation in P + M-treated mice. Western blot analysis revealed that glybenclamide significantly decreased the expressions of both monomeric (Kruskal Wallis *H* test: *H*(2) = 8.187, *p* = 0.017) and oligomeric α-synuclein (*F*_(2,15)_ = 14.788, *p* < 0.01) in the brainstem of mice treated with P + M (Fig. 8c and d). Notably, although no other band was detected, we still cannot exclude the possibility that glybenclamide could reduce expressions of dimer, trimer, or tetramer of α-synuclein. The reasons might be due to the low levels of these forms of α-synuclein that beyond our detect limitation in our conditions.

## Discussion

**Fig. 9:**
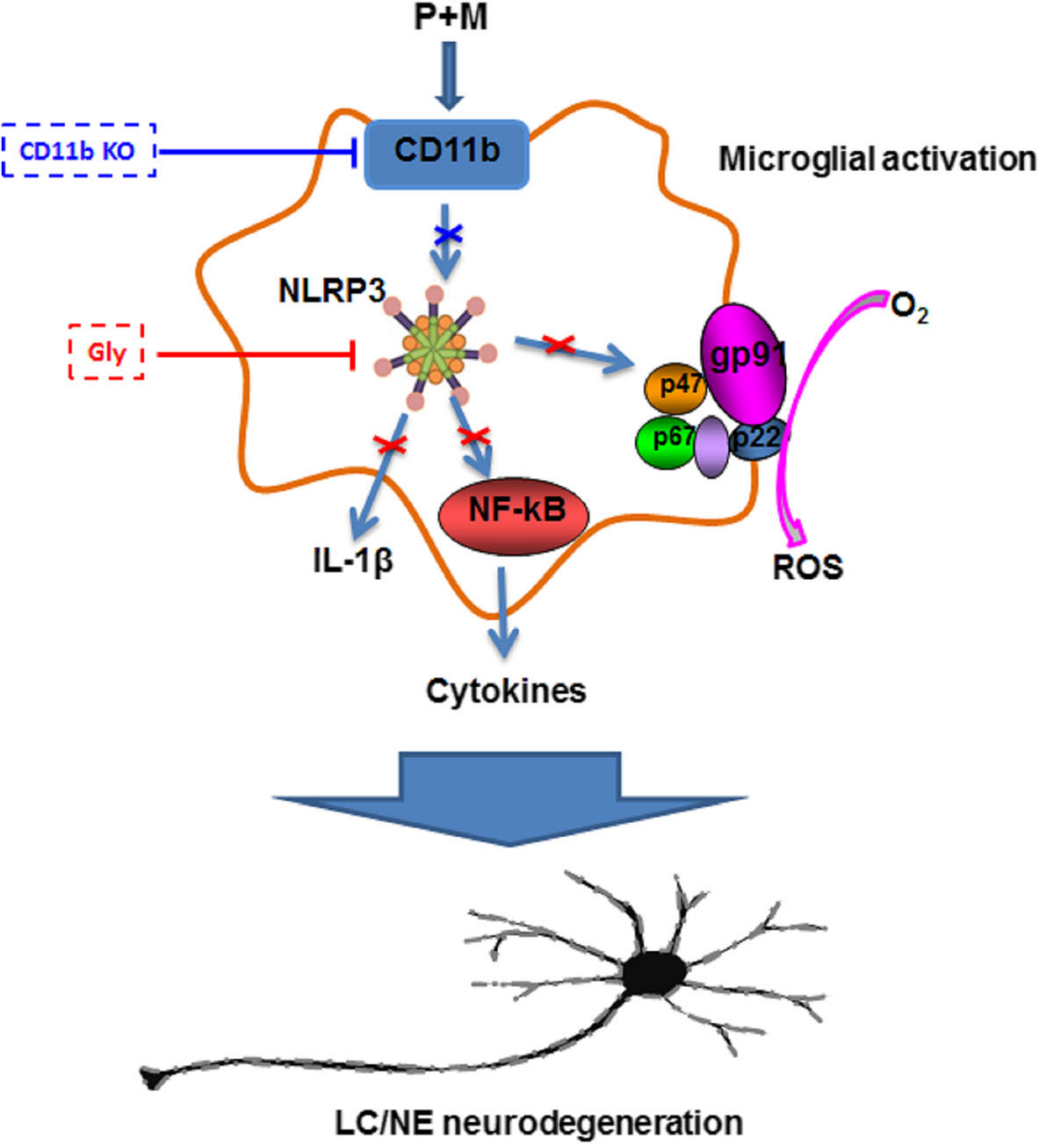
Proposed model showing how CD11b mediates P + M-induced LC/NE neurodegeneration. P + M exposure in mice elevates CD11b expression in the LC. Genetic deletion of CD11b attenuates P + M-induced LC/NE neurodegeneration, indicating a CD11b-dependent pathway. Our results show that CD11b mediates NLRP3 inflammasome activation in P + M-treated mice. Inhibition of NLRP3 inflammasome by glybenclamide suppresses P + M-induced microglial proinflammatory activation and NADPH oxidase activation as well as ROS production, which are associated with LC/NE neuroprotection

Beyond nigral dopaminergic neurodegeneration, patients with PD also display neuronal damage in multiple brain regions, including LC [1, 4]. The degeneration of LC/NE neurons is well-documented in PD, which might be one of the reasons for the appearance of non-motor symptoms in patients [1]. Consistent with that of PD patients, we recently found the loss of LC/NE neurons in P + M and LPS-intoxicated mice [7, 22, 30], two widely used mouse PD models. Furthermore, pre-lesion of LC/NE neurons by DSP-4 significantly exacerbates neurodegeneration in the subsantia nigra, hippocampus, and cortex, which were associated with aggravated motor and non-motor symptoms, such as learning and memory deficits, in these two models [40]. However, the potential mechanisms for LC/NE neurodegeneration in PD remained to be determined. In this study, by using P + M-induced mouse PD model, we found that CD11b-mediated NLRP3 inflammasome activation and subsequent microglial proinflammatory activation as well as oxidative damage contributed to LC/NE neurodegeneration in PD (Fig. 9).

Microglia-mediated neuroinflammation might be involved in the regulatory effects of CD11b on LC/NE neurodegeneration. Microglial activation and accumulation of proinflammatory factors are observed in the brain, including LC region, in PD patients [41]. In vitro studies showed that depletion of microglia in primary midbrain neuron-glia culture is able to attenuate dopaminergic neurodegeneration induced by LPS, MPP^+^, rotenone, and paraquat; four toxins often used to create PD models [15, 42, 43]. We recently reported that microglia are activated in the LC in P + M-treated mice [22, 30]. Inhibition of microglia-mediated neuroinflammation by taurine, a sulfur-containing free amino acid, ameliorates the loss of LC/NE neurons in this mouse PD model [30], indicating a detrimental role of microglia-mediated neuroinflammation in LC/NE neurodegeneration in PD. In the present study, we found that CD11b deficiency significantly suppressed microglial activation and gene expressions of proinflammatory factors in the brainstem in P + M-treated mice, suggesting that CD11b mediates P + M-induced LC/NE neurodegeneration by augmenting microglia-mediated neuroinflammation. Similar to our study, Gao et al. reported that genetic ablation of CD11b mitigates HMGB1 microglial activation, NF-κB activation, and production of multiple inflammatory and neurotoxic factors in primary neuron-glial cultures [15]. Furthermore, inhibition of CD11b by a blocking antibody also blocks diesel exhaust particle-induced microglial activation, H_2_O_2_ production, and neurodegeneration in vitro [15].

Mechanistically, the most critical question to address is how CD11b regulates microglia-mediated neuroinflammation. NLRP3 inflammasome is an intracellular multiprotein complex composed of the NLRP3 scaffold, apoptosis-associated speck-likeprotein (ASC) adaptor, and procaspase-1. NLRP3 inflammasome can sense damage associated molecular patterns (DAMPs) and become activated, leading to activation of caspase-1 and secretion of some proinflammatory cytokines, such as IL-1β and IL-18 [44–46]. Strong evidence suggested that the activation of NLRP3 inflammasome is a key factor to regulate microglial activation in neurodegenerative diseases [47]. Heneka et al. reported that genetic inactivation of NLRP3 attenuates microglial activation and proinflammatory factors production in APP/PS1 mouse AD model [48]. In MPTP-induced mouse PD model, Lee et al. found that genetic inactivation of NLRP3 abolishes MPTP-induced microglial activation, recruitment, and IL-1β production in the subsantia nigra of mouse brain [49]. Furthermore, pharmacological inhibition of NLRP3 inflammasome also displays potent inhibitory effects on microglial activation in 6-OHDA and α-synuclein rodent PD models [33]. Consistent with these reports, NLRP3 inflammasome activation was observed in P + M-treated mice. CD11b deficiency greatly reduced P + M-induced activation of NLRP3 inflammasome. Furthermore, inhibition of NLRP3 inflammasome by glybenclamide abrogated P + M-induced microglial proinflammatory activation. Glybenclamide also prevented oxidative stress and expressions of NOX2 and iNOS, two enzymes responsible for superoxide and RNS production, respectively, during neuroinflammation. More importantly, inhibition of NLRP3 inflammasome by glybenclamide was associated ameliorated LC/NE neurodegeneration and α-synuclein aggregation in P + M-injected mice. These results suggest that NLRP3 inflammasome may be a key factor for CD11b-mediated microglial activation and subsequent LC/NE neurodegeneration. Notably, the mechanisms behind how CD11b regulates NLRP3 inflammasome activation remain to be investigated in the present study, further research focusing on this point should be guaranteed in the future.

## Conclusions

In summary, our findings reveal a novel role of CD11b in LC/NE neurodegeneration through NLRP3 inflammasome-dependent microglial proinflammatory activation and oxidative stress in a two pesticide-induced mouse PD model, providing a novel insight for the immunopathogenesis of LC/NE neuronal damage in PD. Our study also suggests that CD11b might be a promising target for the development of therapeutic agents combating LC/NE neurodegeneration in patients suffering from this disorder.

## Data Availability

All data generated or analyzed during this study are included in this published article [and its supplementary information files].

## Abbreviations

Aβ: β-amyloid
AD: Alzheimer disease
ASC: Apoptosis-associated speck-likeprotein
CNS: Central nervous system
DAMPs: Damage associated molecular patterns
DSP-4: *N*-(2-Chloroethyl)-*N*-ethyl-2-bromobenzylamine
4-HNE: 4-Hydroxynonenal
Iba-1: Ionized calcium binding adaptor molecule-1
IL-1β: Interleukin-1β
LC/NE: Locus coeruleus noradrenergic
LPS: Lipopolysaccharide
MCAO: Middle cerebral artery occlusion
MDA: Malondialdehyde
MPTP: 1-Methyl-4-phenyl-1,2,3,6-tetrahydropyridine
NLRP3: NLR family pyrin domain containing 3
PD: Parkinson’s disease
PMA: Phorbol myristate acetate
TH: Tyrosine hydroxylase
THir: TH-immunoreactive
TNFα: Tumor necrosis factor α
WT: wild type
